# Which cancer survivors are at risk for a physically inactive and sedentary lifestyle? Results from pooled accelerometer data of 1447 cancer survivors

**DOI:** 10.1186/s12966-019-0820-7

**Published:** 2019-08-16

**Authors:** M. G. Sweegers, T. Boyle, J. K. Vallance, M. J. Chinapaw, J. Brug, N. K. Aaronson, A. D’Silva, C. S. Kampshoff, B. M. Lynch, F. Nollet, S. M. Phillips, M. M. Stuiver, H. van Waart, X. Wang, L. M. Buffart, T. M. Altenburg

**Affiliations:** 1 Department of Epidemiology and Biostatistics, Amsterdam Public Health research institute, Amsterdam UMC, Vrije Universiteit Amsterdam, Amsterdam, The Netherlands; 2 Cancer Center Amsterdam, Amsterdam UMC, Vrije Universiteit Amsterdam, Amsterdam, The Netherlands; 3 Australian Centre for Precision Health, School of Health Sciences, University of South Australia Cancer Research Institute, Adelaide, Australia; 4 Faculty of Health Disciplines, Athabasca University, Athabasca, Canada; 5 Department of Public and Occupational Health, Amsterdam Public Health research institute, Amsterdam UMC, Vrije Universiteit Amsterdam, Amsterdam, The Netherlands; 6 National Institute for Public Health and the Environment (RIVM), Bilthoven, The Netherlands; 7 Division of Psychosocial Research and Epidemiology, Netherlands Cancer Institute, Amsterdam, The Netherlands; 8 Faculty of Kinesiology, University of Calgary, Calgary, Alberta Canada; 9 Department of Medical Oncology, Amsterdam UMC, Vrije Universiteit Amsterdam, Amsterdam, The Netherlands; 10 Cancer Epidemiology Division, Cancer Council Victoria, Melbourne, Australia; 11 Centre for Epidemiology and Biostatistics, Melbourne School of Population and Global health, The University of Melbourne, Melbourne, Australia; 12 Physical Activity Laboratory, Baker Heart and Diabetes Institute, Melbourne, Australia; 13 Department of Rehabilitation, Amsterdam Movement Sciences institute, Amsterdam UMC, University of Amsterdam, Amsterdam, The Netherlands; 14 Department of Behavioural Medicine, Northwestern University, Chicago, USA; 15 Center for Quality of Life, Netherlands Cancer Institute, Amsterdam, The Netherlands; 16 Department of Anesthesiology, University of Auckland, Auckland, New Zealand

**Keywords:** Physical activity, Sedentary time, Cancer survivors, Profile analysis, Activity profiles

## Abstract

**Background:**

Physical activity has beneficial effects on the health of cancer survivors. We aimed to investigate accelerometer-assessed physical activity and sedentary time in cancer survivors, and describe activity profiles. Additionally, we identify demographic and clinical correlates of physical activity, sedentary time and activity profiles.

**Methods:**

Accelerometer, questionnaire and clinical data from eight studies conducted in four countries (*n* = 1447) were pooled. We calculated sedentary time and time spent in physical activity at various intensities using Freedson cut-points. We used latent profile analysis to identify activity profiles, and multilevel linear regression analyses to identify demographic and clinical variables associated with accelerometer-assessed moderate to vigorous physical activity (MVPA), sedentary time, the highly active and highly sedentary profile, adjusting for confounders identified using a directed acyclic graph.

**Results:**

Participants spent on average 26 min (3%) in MVPA and 568 min (66%) sedentary per day. We identified six activity profiles. Older participants, smokers and participants with obesity had significantly lower MVPA and higher sedentary time. Furthermore, men had significantly higher MVPA and sedentary time than women and participants who reported less fatigue had higher MVPA time. The highly active profile included survivors with high education level and normal body mass index. Haematological cancer survivors were less likely to have a highly active profile compared to breast cancer survivors. The highly sedentary profile included older participants, males, participants who were not married, obese, smokers, and those < 12 months after diagnosis.

**Conclusions:**

Cancer survivors engage in few minutes of MVPA and spend a large proportion of their day sedentary. Correlates of MVPA, sedentary time and activity profiles can be used to identify cancer survivors at risk for a sedentary and inactive lifestyle.

**Electronic supplementary material:**

The online version of this article (10.1186/s12966-019-0820-7) contains supplementary material, which is available to authorized users.

## Introduction

Previous reviews and meta-analyses of randomized controlled trials have demonstrated beneficial effects of physical activity on a variety of physical and psychosocial health outcomes in cancer survivors [1–4]. In addition, higher levels of physical activity has been associated with lower risk of disease recurrence and mortality in breast, [5–7] colon [7, 8] and prostate cancer survivors [9]. Sedentary time, defined as any waking behaviour in a sitting, lying or reclined posture with low energy expenditure, [10, 11] has been associated with adverse health outcomes in cancer survivors such as weight gain, cardiovascular disease, and also increased mortality in patients diagnosed with colorectal cancer [12–14]. Recent studies reported that few cancer survivors engage in regular physical activity of sufficient duration and intensity and survivors spent the majority of their waking time in sedentary pursuits [15, 16].

Previous studies in non-Hodgkin lymphoma, breast and colon cancer survivors suggested that levels of accelerometer assessed physical activity and sedentary time may differ between survivors with different demographic and clinical characteristics [17–20]. These studies showed that older age, higher body mass index (BMI), smoking and being unemployed were associated with lower moderate to vigorous physical activity (MVPA) levels [17–19]. Multiple comorbidities, a higher disease stage, smoking and higher BMI have been associated with higher sedentary time [17, 18, 20]. However, differences in accelerometer processing techniques, statistical analysis methods and available correlates hinder comparison between studies [21]. Furthermore, given these studies focused on specific groups of patients with homogeneous tumour types, it was not possible to examine cancer type as a potential correlate of physical activity and sedentary time.

In this study we investigate levels of accelerometer assessed physical activity and sedentary time. To acknowledge both physical activity and sedentary time, Thompson et al. suggested to describe activity profiles rather than a single dimension of physical activity or sedentary time [22]. Therefore, we describe activity profiles based on multiple indicators of physical activity and sedentary time. This study is the first to utilize a large, pooled dataset including cancer survivors with different types of cancer, using uniform accelerometer-derived measures of these behaviours, based on pooled data from different studies. In addition, we investigate demographic and clinical correlates of MVPA, sedentary time and activity profiles. This information may help to identify survivors who are more likely to engage in unhealthy levels of physical activity and sedentary time and may assist in developing and targeting interventions for patients with a specific activity profile.

## Method

### Study design

We pooled demographic, clinical and accelerometer data from cancer survivors who had completed cancer treatment, collected in eight studies from Australia, Canada, the Netherlands and the United States. Full details of individual study designs and inclusion criteria have been described previously [16, 17, 23–28]. A summary of study characteristics and data collection procedures is presented in Table 1. Data from participants were included in the current analyses when demographic, clinical and accelerometer data were available and when participants did not receive a physical activity intervention during data collection.

**Table 1:** Study characteristics

Study	Country	Cancer type	Number of participants	Age, mean (SD)	Sex (% female)	Inclusion criteria	Study design	Data collection		
								Demographic and clinical information	Actigraph accelerometer	Fatigue
Boyle, 2016 [16]	Australia	Breast	252	60.4 (10.5)	100	- Being female - Aged 18–80 years of age at time of diagnosis - Being 1–3 years post-diagnosis - Residing in Western-Australia at time of diagnosis - Not diagnosed with another cancer - Completed cancer treatment	Cross-sectional	Self-report	GT3X-Plus	FACT-Fatigue
Boyle, 2017 [17]	Australia	Non-Hodgkin lymphoma	156	62.5 (12.8)	47.7	- Histologically confirmed NHL diagnosis - Residing in Western-Australia at time of study - Not diagnosed with another cancer - Completed cancer treatment	Cross-sectional	Self-report	GT3X	FACT-Fatigue
D’Silva, 2018 [27]	Canada	Lung	121	71.2 (8.9)	57.0	- Previous clinical and/or pathological diagnosis of NSCLC - Not currently receiving any treatment for lung cancer or any other cancer - Not living in a hospice or long-term care - Age ≥ 18 years - Ability to read and write English	Cross-sectional	Self-report (co-morbidities, smoking status, demographic characteristics) and Glans-Look Lung Cancer Database (clinical characteristics)	GT3X-Plus	FACT-Fatigue
Kampshoff, 2015 [24]	The Netherlands	Mixed	232	54.1 (10.9)	79.7	- Histologically confirmed breast, colon, ovarian, cervix or testis cancer or lymphomas - No indication of recurrent or progressive disease - Aged ≥18 years - Able to perform basic activities of daily living - No cognitive disorders of severe emotional instability - No other serious disease that might hamper patients’ ability to carry out exercise - Ability to understand the Dutch language	Multicentre RCT (Baseline data; 4–6 weeks after completion of primary treatment)	Self-report (socio-demographic variables) and medical records (clinical characteristics)	Actitrainer	MFI
Persoon, 2017 [25]	The Netherlands	Haematological	82	52.7 (10.1)	37.8	- Treated with auto-stem cell transplantation for multiple myeloma or lymphoma, - Able to undergo exercise testing and participate in an exercise intervention	Multicenter RCT (Baseline data; 6–14 weeks after transplantation)	Self-report (socio-demographic characteristics) and medical records (clinical characteristics)	Actitrainer	MFI
Phillips, 2015 [28]	United States	Breast	412	56.7 (9.2)	100	- Age ≥ 18 years - Prior breast cancer history - English-speaking - Access to the Internet	Prospective longitudinal study (Baseline data from a subset of patients who participated in an on-line questionnaire study)	Self-report	GT1M	FSI
Vallance, 2015 [26]	Australia/ Canada	Colon	156	64.5 (9.8)	48.7	- Histologically confirmed stage I-III colon cancer - Aged 18–80 years of age - Completed cancer treatment - English speaking	Cross-sectional	Self-report	GT3X-Plus	FACT-Fatigue
Van Waart, 2017 [23]	The Netherlands	Breast/ colon	36	52.9 (8.7)	97.2	- Histologically confirmed primary breast or colon cancer - No orthopaedic, cardiovascular or cardiopulmonary conditions - Not suffering from malnutrition, serious psychiatric or cognitive problems - Ability to understand the Dutch language	Multicentre RCT (Follow up data from patients randomized to the control group)	Self-report (socio-demographic variables) and medical records (clinical characteristics)	Actitrainer	MFI

*FACT-fatigue* Functional assessment of cancer therapy – fatigue questionnaire, *MFI* Multidimensional fatigue inventory, *FSI* fatigue symptom inventory, *NHL* Non-Hodgkin lymphoma, *NSCLC* non-small-cell lung cancer, *RCT* randomized controlled trial, *SD* standard deviation

### Accelerometer data reduction

Accelerometer data during waking hours were collected for five [25] or seven [16, 17, 23, 24, 26–28] consecutive days with ActiGraph accelerometers (Florida, USA) and processed in a customized software program developed in R version 3.2.5, [29] using the vertical axis, standard filtering and 60-s epochs. Non-wear time was defined as ≥60 min of consecutive zero counts and was excluded during data processing [30, 31]. Valid days were defined as days with at least 600 min of wear time. According to Trost et al., three to five valid days are necessary to calculate a reliable estimate for physical activity in adults [32]. Because our data showed significant differences in time estimates between week- and weekend days, we have included patients with at least three valid weekdays and one valid weekend day [30, 33]. Activity counts were categorized as sedentary (< 100 counts per minute (cpm)), light-intensity physical activity (100- < 1952 cpm) and MVPA (≥1952 cpm) [34, 35].

As total physical activity has been associated with health benefits, [36] we calculated estimates of total activity counts (in counts per day). Additionally, we calculated estimates of total volumes (minutes per valid day) of sedentary behaviour, light physical activity and MVPA as MVPA may have greater benefits compared to light physical activity [37].

Although the American College of Sports Medicine (ACSM) physical activity guidelines for cancer survivors no longer recommend accumulating MVPA in bouts of at least ten minutes, other international guidelines (e.g. World Health Organization) currently include this bout criterion [38, 39]. Therefore, MVPA accumulated in bouts of at least ten consecutive minutes, with allowance for an interruption of < 10% and an absolute tolerance of three consecutive minutes, was still examined for comparison with other studies. Since laboratory studies have shown that interrupting sedentary time every 20 min with light intensity walking for 2 min reduces glucose levels, [40, 41] we calculated time in sedentary bouts of 20 min or more, without allowance for interruptions [42]. Cancer survivors often have a lower peak oxygen consumption compared to the general population [24, 43] and currently available cut-points might underestimate relative physical activity intensities for participants with low peak oxygen consumption [44]. Therefore, we also estimated total volume and time accumulated in bouts of at least ten minutes of light and total physical activity. Furthermore, we calculated the average cpm in light physical activity and the 75th percentile of cpm in light physical activity as indicators of the intensity of light physical activity. Finally, we calculated the number of bouts in sedentary time, light intensity physical activity, total physical activity and MVPA per valid day. Table 2 contains a complete list of accelerometer variables used in this study.

**Table 2:** Demographic and clinical characteristics, physical activity and sedentary time of participants

	Participants (*n* = 1447)	
*Demographic*		
Age, mean (SD) years	59.3 (11.4)	
Sex, n (%)		
Women	1134 (78.4)	
Marital status, n (%)		
Married	1137 (78.6)	
Education level, n (%)		
Low	165 (11.4)	
Middle	696 (48.1)	
High	571 (39.9)	
Employment, n (%)		
Unemployed	181 (12.5)	
Employed	700 (48.4)	
Retired	470 (32.5)	
Missing	96 (6.6)	
*Clinical*		
BMI, mean (SD) kg/m^2^	26.1 (5.0)	
Cancer Type, n (%)		
Breast	844 (58.3)	
Testicular	5 (0.3)	
Haematological	259 (17.9)	
Colorectal	205 (14.2)	
Gynaecological	13 (0.9)	
Lung	121 (8.4)	
Treatment, n (%)		
No treatment/only surgery	254 (17.6)	
Surgery + chemotherapy	489 (33.8)	
Surgery + radiotherapy	218 (15.1)	
Surgery + chemotherapy + radiotherapy	432 (29.9)	
Missing	54 (3.6)	
Comorbidities, n (%)		
None	562 (38.8)	
One or more	835 (57.7)	
Missing	50 (3.5)	
Time since diagnosis, median (IQR) months	46.6 (15.3–51.3)	
Fatigue, mean (SD)		
FACIT-fatigue	42.2 (9.2)	
MFI-general fatigue	12.6 (3.9)	
FSI-disruption index	2.0 (2.0)	
	Mean (SD)	Wear time
*Physical activity and sedentary time*		
Accelerometer wear time per day		
Minutes	864.5 (70.3)	100%
Sedentary time per day		
Minutes	568.1 (91.8)	66%
Time in bouts of ≥20 min	261.8 (102.8)	30%
Number of bouts, n	7.2 (2.4)	
Light-intensity physical activity per day		
Minutes	270.0 (77.5)	31%
75th percentile, counts	709.2 (124.7)	
Moderate-to-vigorous intensity physical activity per day		
Minutes	26.4 (19.8)	3%
Time in bouts of ≥10 min	3.9 (8.6)	0.5%
Number of bouts, n	0.2 (0.3)	
Total physical activity per day		
Minutes	296.4 (89.3)	34%
Time in bouts of ≥10 min	139.4 (86.8)	16%
Number of bouts, n	6.6 (3.3)	
Total counts		
Average counts per day	243494 (118368)	
Average counts per minute	281 (132)	

*BMI* body mass index, *FACIT* functional assessment of chronic illness therapy, *FSI* fatigue symptom inventory, *kg* kilogram, *m* meter, *MFI* multidimensional fatigue inventory, *n* number of participants, *SD* standard deviation, *IQR* interquartile range

### Potential demographic and clinical correlates

All studies used self-report questionnaires to collect demographics variables, including age, sex, marital status (dichotomized into not married – never married; separated; widowed or divorced; and married - de facto or married), education level (categorized into: low - not completed high school; medium - completed high school, trade school/apprenticeship or some university; and high - completed university or graduate school), employment (categorized as unemployed, part-time/full-time and retired) and smoking status (dichotomized into current smoker and non-smoker). BMI was calculated from (self-reported) weight and height. Clinical variables were collected using questionnaires [16, 17, 26, 28] or medical records [23–25] and included cancer type (categorized into haematological, gastrointestinal, gynaecological, breast, lung and testicular cancer), type of treatment (categorized into no treatment/only surgery, surgery + chemotherapy, surgery + radiotherapy and surgery + chemotherapy + radiotherapy), time since diagnosis and the presence of comorbidities (dichotomized into no comorbidities and one or more comorbidities, including heart disease, high blood pressure, diabetes, high blood cholesterol, osteoporosis, asthma, neurological disease, gastrointestinal disease, depression, anxiety disorder, degenerative disease and migraine). Fatigue was assessed using the functional assessment of cancer therapy (FACT)-fatigue questionnaire [45] in the studies conducted in Australia and Canada, [16, 17, 26, 27] the general fatigue score from the multidimensional fatigue inventory (MFI) [46] in the Netherlands [23–25] and the disruption index from the fatigue symptom inventory (FSI) [47] in the United States [28]. Fatigue scores were pooled after transformation into standardized or ‘z-scores’ which were calculated by subtracting the mean score of each questionnaire from the individual scores at baseline and dividing the result by the mean standard deviation. To better interpret associations between continuous variables and physical activity and sedentary time, estimates for relevant subgroups are presented. Age was categorized as < 45 years, 45- < 55 years, 55- < 65 years, 65- < 75 years and ≥ 75 years. BMI was categorized as underweight (< 18.5 kg/m^2^), normal weight (18.5 to < 25 kg/m^2^), overweight (25 to < 30 kg/m^2^) and obese (≥30 kg/m^2^). Time since diagnosis was categorized as < 12 months, 12 to < 36 months, 36 to < 120 months and ≥ 120 months. Fatigue was categorized based on the z-scores from the study population as ‘average fatigue’, ≤0.5 standard deviation (SD) below average and ≥ 0.5 SD above average, as these cut-points resulted in three groups of roughly equal size.

### Statistical analyses

**Fig. 1:**
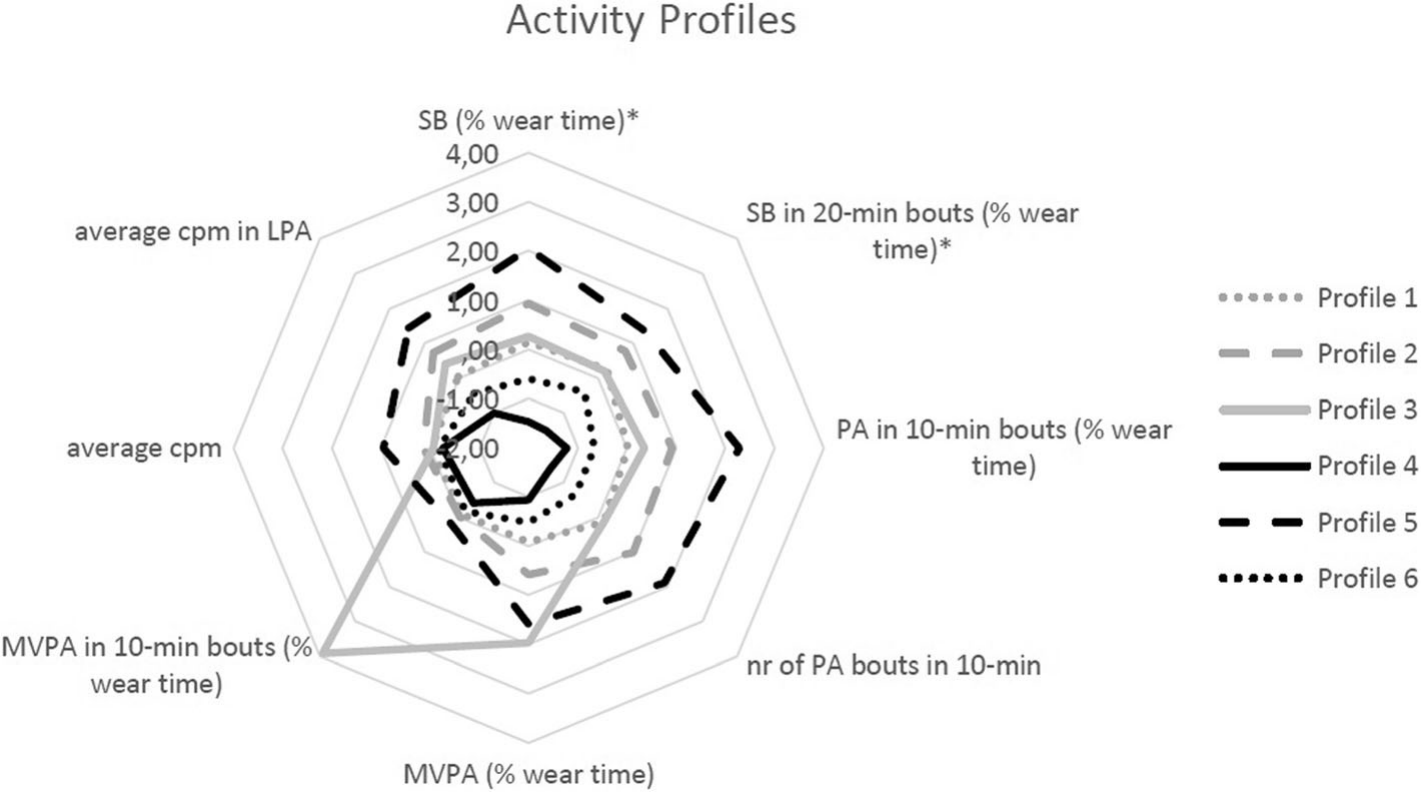
Standardized profile means (z-scores). Cpm = counts per minute, LPA = light physical activity, MVPA = moderate-to-vigorous physical activity, MVPA-bout = MVPA time in bouts of 10 min, nr = number, SB = sedentary behaviour, SB-bout = sedentary behavour in bouts of 20 min. Note: SB (% wear time) and SB in 20-min bouts (% wear time) have switched signs to ‘higher is better’. Profile 1: the average profile, Profile 2: the high potential profile, Profile 3: the highly active profile, Profile 4: the highly sedentary profile, Profile 5: the sufficiently active profile, Profile 6: the insufficiently active profile

Activity profiles were identified with latent profile analysis. We initially considered all 14 physical activity and sedentary time indicators (Table 2). Due to high correlations between some of these variables (total sedentary time and total physical activity time; total sedentary time and total light physical activity time; time in sedentary bouts and number of sedentary bouts; 75th percentile of cpm in light physical activity and average cpm in light physical activity; average counts per day and average cpm; time in MVPA bouts and number of MVPA bouts), we reduced this to eight indicators (Fig. 1). Total MVPA time, time in MVPA bouts, time in physical activity bouts, total sedentary time and time in sedentary bouts were included as percentage of total wear time (%wear time). The optimal number of activity profiles was based on a combination of Bayesian information criterion (BIC), global entropy and clinical relevance, [48] and was set at a maximum of six. Each participant was fitted into the activity profile for which they had the highest probability of belonging to. Descriptive statistics were used to summarize the means and standard deviations of the eight indicators in each of the identified activity profiles. To visualize differences between profiles, standardized profile means (z-scores) of the indicators were calculated (Fig. 1).

We performed multivariable multilevel linear regression analyses to assess correlates of MVPA and sedentary time, both for total time and time accumulated in bouts. The associations between each of the hypothesized correlates and MVPA or sedentary time were estimated in separate models in order to avoid the Table Two Fallacy (i.e. when effect estimates for multiple variables in the same model are all incorrectly interpreted as total effect estimates) [49]. A minimal sufficient set of confounders was chosen for each correlate using a directed acyclic graph (DAG; Additional file 1: Figure S1) [49, 50]. The associations depicted in the DAG were based on the hypothesized causal effects between the variables from previous literature and/or expert opinion, and were derived from input from six researchers (MGS, TB, JV, BL, LB, TA). All models had a two-level structure (1: participant, 2: study) and a random intercept on study level to take into account clustering of participants within studies. All multilevel linear regression analyses were adjusted for accelerometer wear time. We used multilevel linear regression analyses to investigate the associations between each of the demographic and clinical correlates and the (posterior) probability (which could be any proportion between 0 and 1) of belonging to the two most extreme profiles, i.e. the profile with highest sedentary time (highly sedentary profile) and the profile with highest MVPA (highly active profile) with the minimal sufficient adjustment set of potential confounders from the DAG.

## Results

### Participant characteristics

Accelerometer data were available for 1623 cancer survivors and data of 1447 participants met the criteria of three valid weekdays and one valid weekend day. Participants (78% females) were, on average, 59 (SD 11) years old, 40% were highly educated and the mean BMI was 26.2 (SD 6.3) kg/m^2^ (Table 2).

### Physical activity, sedentary time and activity profiles

Participants wore the accelerometer for, on average, 14.4 (SD 1.2) hours per day, of which they spent, on average, 26 (SD 20) minutes per day in MVPA and 9.5 (SD 1.5) hours per day sedentary. Participants accumulated on average 3.9 (SD 8.6) minutes per day in MVPA bouts and 4.3 (SD 1.7) hours per day in sedentary bouts. Based on BIC, global entropy and clinical relevance, six activity profiles were identified. Table 3 presents mean values of the indicators of the different activity profiles and the demographic and clinical characteristics of participants that fit within that profile. Profile 1 – the average profile, including 29% of participants – was characterized by average estimates of sedentary time (64%), physical activity bouts (16%) and total MVPA (3%). Profile 2 – the high potential profile, 18% of participants - was characterized by the second lowest sedentary time (57%) and second highest time in physical activity bouts (25%). Profile 3 – the highly active profile, 3% of participants – had highest total MVPA (7%) and highest time in MVPA bouts (4%). Profile 4 – the highly sedentary profile, 14% of participants – was characterized by the highest sedentary time (80%) and lowest MVPA time (0%). Profile 5 – the sufficiently active profile, 18% of participants – had lowest sedentary time (46%), highest time in physical activity bouts (39%) and second highest MVPA time (7%) and time in MVPA bouts (1%). Profile 6 – the insufficiently active profile, 28% of participants – was characterized by the second highest sedentary time (72%), and second lowest MVPA time (2%).

**Table 3:** Indicators and demographic and clinical characteristics of activity profiles

	Profile 1 Average profile	Profile 2 High potential profile	Profile 3 Highly active profile	Profile 4 Highly sedentary profile	Profile 5 Sufficiently active profile	Profile 6 Insufficiently active profile
Number of participants	414	267	45	204	110	407
Probability (median, range)	1.0 (0.5–1.0)	1.0 (0.5–1.0)	1.0 (0.6–1.0)	1.0 (0.5–1.0)	1.0 (0.6–1.0)	1.0 (0.5–1.0)
*Indicators of activity profiles*						
Average counts (counts/minute, SD)	279.2 (60.5)	377.7 (79.9)	515.8 (102.1)	123.3 (41.4)	516.4 (108.0)	207.1 (57.2)
Total sedentary time (% wear time, SD)	64.4 (2.8)	56.8 (3.3)	63.3 (4.9)	80.0 (3.5)	45.8 (4.3)	71.7 (2.7)
Sedentary time in bouts (% wear time, SD)	27.3 (7.1)	21.1 (6.7)	28.1 (7.3)	48.3 (8.8)	14.1 (5.5)	35.2 (7.6)
Total PA in bouts (% wear time, SD)	16.2 (2.7)	25.1 (3.6)	19.5 (5.1)	4.0 (2.2)	38.5 (5.8)	9.5 (2.5)
Number of PA bouts (n, SD)	7.1 (1.1)	10.0 (1.2)	7.1 (2.0)	2.0 (1.0)	12.8 (2.0)	4.4 (1.0)
Average counts in LPA (counts/min, SD)	375.0 (60.5)	421.7 (51.8)	399.8 (41.6)	305.6 (41.4)	469.5 (61.1)	342.9 (44.6)
Total MVPA (% wear time, SD)	2.9 (1.3)	4.3 (1.8)	7.4 (1.9)	0.9 (0.7)	6.5 (2.5)	2.0 (1.2)
MVPA bouts (% wear time, SD)	0.4 (0.1)	0.4 (0.1)	4.2 (1.6)	0.0 (0.0)	0.6 (0.1)	0.2 (0.1)
*Demographic and clinical characteristics*						
Age, mean (SD) years	59.3 (10.9)	57.5 (10.9)	56.0 (9.3)	62.1 (12.5)	58.6 (10.8)	59.7 (11.8)
Sex, % (n)						
Female	81.2 (336)	83.1 (222)	80.0 (36)	66.7 (136)	75.5 (83)	78.9 (321)
Marital status, % (n)						
Married	80.7 (334)	82.8 (221)	86.7 (39)	70.6 (144)	74.5 (82)	77.9 (317)
Education, % (n)						
Low	9.2 (38)	12.7 (34)	2.2 (1)	11.3 (23)	20.9 (23)	11.3 (46)
Middle	48.6 (201)	52.4 (140)	22.2 (10)	49.5 (101)	50.9 (56)	46.2 (188)
High	41.3 (171)	33.7 (90)	75.6 (34)	38.7 (79)	26.4 (29)	41.3 (168)
Employment, % (n)						
Unemployed	11.4 (47)	15.7 (42)	2.2 (1)	11.3 (23)	13.6 (15)	13.0 (53)
Employed	53.4 (221)	53.6 (143)	62.2 (28)	26.5 (54)	51.8 (57)	48.4 (197)
Retired	31.4 (130)	29.6 (79)	31.1 (14)	40.7 (83)	31.8 (35)	31.7 (129)
BMI, mean (SD) kg/m^2^	26.2 (4.7)	26.2 (4.6)	23.3 (2.8)	26.2 (5.9)	26.2 (4.9)	26.3 (5.2)
Smoking status, % (n)						
Non-smoker	94.2 (390)	95.5 (255)	100 (45)	84.3 (172)	94.5 (104)	92.9 (378)
Diagnosis, % (n)						
Breast	62.8 (260)	61.0 (163)	71.1 (32)	45.1 (92)	52.7 (58)	58.7 (239)
Gastrointestinal	12.3 (51)	20.2 (54)	15.6 (7)	6.9 (14)	18.2 (20)	14.5 (59)
Haematological	16.2 (67)	15.0 (40)	8.9 (4)	27.9 (57)	27.3 (30)	15.0 (61)
Testicular	0.5 (2)	0.4 (1)	0.0 (0)	0.5 (1)	0.0 (0)	0.2 (1)
Lung	7.5 (31)	2.2 (6)	4.4 (2)	19.6 (40)	1.8 (2)	9.8 (40)
Gynaecological	0.7 (3)	1.1 (3)	0.0 (0)	0.0 (0)	0.0 (0)	1.7 (7)
Treatment, % (n)						
No treatment/only surgery	17.6 (73)	17.6 (47)	13.3 (6)	17.6 (36)	20.9 (23)	17.0 (69)
Surgery + chemotherapy	34.3 (142)	31.5 (84)	37.8 (17)	39.7 (81)	34.5 (38)	31.2 (127)
Surgery + radiotherapy	15.0 (62)	15.7 (42)	17.8 (8)	16.7 34)	13.6 (15)	14.0 (57)
Surgery + radiotherapy + chemotherapy	28.3 (117)	30.7 (82)	31.1 (14)	25.0 (51)	26.4 (29)	34.2 (139)
Comorbidities, % (n)						
No comorbidities	39.9 (41)	49.4 (132)	51.1 (23)	25.5 (52)	50.0 (55)	33.2 (135)
Time since diagnosis, median (IQR) months	33.0 (15.1–48.6)	30.4 (17.6–40.0)	39.0 (15.0–79.0)	33.5 (10.0–75.8)	32.5 (23.2–39.2)	32.0 (14.6–68.0)
Fatigue, mean (SD) z-score	−0.1 (1.0)	−0.1 (1.0)	−0.3 (0.9)	0.3 (1.1)	−0.1 (0.9)	0.1 (1.0)

*BMI* body mass index, *kg* kilogram, *m* meter, *min* minute, *n* number of participants, *SD* standard deviation, *IQR* interquartile range

### Correlates of sedentary time

Sedentary time was significantly higher among older participants, males and participants with obesity (Table 4). Sedentary time in bouts was significantly higher among older participants, males, participants with overweight or obesity, participants treated with surgery, radiotherapy and chemotherapy and participants with higher than average fatigue (Table 4).

**Table 4:** Demographic and clinical correlates of sedentary time and MVPA and sedentary and MVPA bouts

	Confounders^a^	Sedentary time, minutes (95% CI)	Sedentary bouts, minutes (95% CI)	MVPA, minutes (95% CI)	MVPA bouts, minutes (95% CI)
Age (years)					
< 45	–	reference	reference	reference	reference
45 to < 55		4.8 (−9.8;19.4)	12.4 (−6.3;31.1)	−2.6 (−6.1;1.0)	0.1 (−1.79;1.56)
55 to < 65		16.7 (2.8;30.6)*	30.7 (12.9;48.6)*	−7.2 (−10.6;−3.8)*	−0.8 (−2.4;0.8)
65 to < 75		33.6 (18.5;48.7)*	62.5 (43.2;81.8)*	−10.8 (−14.5;−7.2)*	−1.7 (−3.4;0.0)
75 ≥		63.5 (44.8;82.3)*	84.1 (60.2;108.1)*	−21.2 (−25.7;−16.6)*	−3.7 (−5.8;−1.5)*
Sex	–				
Males		reference	reference	reference	reference
Females		−25.1 (−36.4;−13.8)*	−45.0 (−59.4;−30.6)*	−3.5 (−6.3;−0.8)*	0.1 (−1.2;1.3)
Marital status	Age				
Not married		reference	reference	reference	reference
Married		−6.3 (−15.7;3.1)	−4.4 (−16.4;7.7)	2.1 (−0.2;4.4)	1.1 (0.0;2.1)
Education level	Age, sex				
Low		reference	reference	reference	reference
Middle		9.5 (−3.1;22.1)	4.6 (−11.4;20.7)	−1.5 (−4.5;1.5)	0.1 (−1.3;1.5)
High		10.7 (−2.8;24.3)	7.8 (−9.4;25.1)	2.5 (−0.8;5.8)	3.2 (1.7;4.7)*
Employment status	Age, sex, marital status, education level, diagnosis, treatment type, comorbidity, fatigue, time since diagnosis				
Unemployed		reference	reference	reference	reference
Employed		0.5 (−12.4;13.4)	−5.9 (−22.1;10.2)	0.2 (−2.9;3.4)	−0.3 (−1.8;1.2)
Retired		4.4 (−10.2;19.0)	11.4 (−6.9;29.7)	2.1 (−1.4;5.7)	1.2 (−0.5;2.8)
Weight status	Age, sex, marital status, education level, smoking status, diagnosis, treatment type, time since diagnosis				
Underweight		0.2 (−26.7;27.1)	−16.0 (−50.4;18.5)	−1.7 (−8.2;4.7)	−0.4 (−3.5;2.7)
Normal weight		reference	reference	reference	reference
Overweight		8.8 (0.0;17.7)	13.3 (2.0;24.6)*	−3.0 (−5.1;−0.9)*	−1.7 (−2.7;−0.6)*
Obese		17.9 (7.4;28.4)*	24.1 (10.7;37.6)*	−7.4 (−9.9;−4.9)*	−3.3 (−4.5;−2.1)*
Smoking status	Age, sex, education level				
Non-smoker		reference	reference	reference	reference
Smoker		26.8 (11.1;42.5)*	12.8 (−7.3;32.8)	−9.4 (−13.1;−5.6)*	−2.7 (−4.5;−0.9)*
Diagnosis	Age, sex, smoking status				
Breast		reference	reference	reference	reference
Gastrointestinal		8.0 (−15.6;31.7)	7.3 (−22.6;37.2)	−3.6 (−9.2;2.0)	−1.2 (−3.1;0.6)
Haematological		20.3 (−11.4;52.1)	24.7 (−14.5;64.0)	−6.0 (−13.2;1.1)	−2.2 (−4.0;−0.4)
Testicular		16.4 (−49.2;82.1)	−25.0 (−108.8;58.9)	−1.2 (−17.1;14.6)	−3.0 (−10.6;4.6)
Lung		36.8 (−63.7;137.3)	5.8 (−102.7;114.2)	−8.4 (−26.2;9.5)	−2.2 (−4.6;0.1)
Gynaecological		−1.9 (−42.9;39.1)	−10.7 (−63.1;41.7)	0.1 (−9.8;9.9)	−3.6 (−8.2;1.1)
Treatment	Age, diagnosis				
No treatment/only surgery		reference	reference	reference	reference
Surgery + chemotherapy		4.8 (−8.1;17.8)	13.4 (−3.1;30.0)	0.2 (−2.9;3.4)	0.7 (−0.7;2.1)
Surgery + radiotherapy		4.4 (−9.7;18.6)	14.1 (−4.0;32.3)	0.9 (−2.6;4.3)	1.4 (−0.2;3.1)
Surgery + radiotherapy + chemotherapy		12.5 (−0.8;25.8)	22.2 (5.2;39.2)*	−0.8 (−4.0;2.4)	−0.3 (−1.7;1.2)
Comorbidity	Age, weight status, smoking status, treatment type				
No comorbidity		reference	reference	reference	reference
One or more comorbidities		8.5 (−0.2;17.3)	10.7 (−0.5;22.0)	−2.1 (−4.1;0.0)	−1.3 (−2.3;−0.3)*
Time since diagnosis (months)	–				
< 12		reference	reference	reference	reference
12 to < 36		−6.9 (−32.0;18.2)	−6.3 (−37.8;25.2)	2.25 (−3.6;8.1)	0.5 (−1.6;2.7)
36 to < 120		−6.0 (−31.2;19.2)	−4.3 (−35.9;27.3)	1.8 (−4.1;7.7)	0.5 (−1.6;2.7)
120 ≥		−5.0 (−33.4;23.5)	−1.9 (−37.8;33.9)	2.2 (−4.5;8.9)	0.8 (−1.8;3.3)
Fatigue	Age, weight status, smoking status, diagnosis, treatment type, comorbidity, time since diagnosis				
< 0.5 SD		−7.6 (−16.9;1.7)	−4.4 (−16.4;7.5)	2.8 (0.6;5.0)*	1.6 (0.5;2.7)*
Mean fatigue		reference	reference	reference	reference
> 0.5 SD		7.6 (−2.4;17.7)	14.0 (1.0;27.0)*	−2.0 (−4.4;0.4)	−0.6 (−1.8;0.6)

*CI* confidence interval, *ref* reference, *SD* standard deviation, * *p* < 0.05

^a^ based on the DAG in Additional file 1: Figure S1

### Correlates of MVPA

MVPA was significantly lower among older participants, females and participants with overweight or obesity. MVPA was significantly higher among participants with lower levels of fatigue (Table 4). The same correlates were found for MVPA in bouts, except for sex (Table 4). Furthermore, we found significantly more time accumulated in MVPA bouts among participants with high education levels and participants without comorbidities.

### Correlates of activity profiles

Participants aged 65 > years, males, smokers, participants who were not married, obese, and participants within the first 12 months after diagnosis had a higher probability of belonging to the highly sedentary profile (Table 5). Participants who were highly educated, and had a normal weight had a higher probability of belonging to the highly active profile. Haematological cancer survivors had a lower probability of belonging to the highly active profile compared to breast cancer survivors.

**Table 5:** Demographic and clinical correlates of the highly sedentary and highly active profile

	Confounders^a^	Highly sedentary profile^#^ (95% CI)	Highly active profile^#^ (95% CI)
Age	–		
< 45 years		reference	reference
45- < 55 years		0.0 (−0.1;0.1)	0.0 (−0.01;0.1)
55- < 65 years		0.0 (−0.02;0.1)	0.0 (−0.03;0.03)
65- < 75 years		0.1 (0.02;0.1)*	−0.002 (−0.04;0.03)
75 ≥ years		0.1 (0.1;0.2)*	−0.03 (−0.1;0.01)
Sex	–		
Males		reference	reference
Females		−0.1 (−0.1;−0.02)*	0.0 (−0.01;0.03)
Marital status	Age		
Not married		reference	reference
Married		−0.1 (−0.1;−0.02)*	0.0 (−0.01;0.03)
Education level	Age, sex		
Low		reference	reference
Middle		0.01 (−0.05;0.06)	−0.01 (−0.02;0.04)
High		−0.01 (−0.06;0.05)	0.1 (0.02;0.08)*
Employment	Age, diagnosis, comorbidity, education, fatigue, marital status, sex, time since diagnosis, treatment type		
Unemployed		reference	reference
Employed		−0.03 (−0.08;0.03)	0.02 (−0.01;0.05)
Retired		0.03 (−0.03;0.09)	0.03 (−0.01;0.06)
BMI	Age, diagnosis, education, marital status, sex, smoking, time since diagnosis, treatment type		
Underweight		0.06 (−0.05;0.2)	−0.04 (−0.1;0.02)
Normal weight		reference	reference
Overweight		0.0 (−0.03;0.04)	−0.02 (−0.04;−0.003)*
Obese		0.04 (0.003;0.09)*	−0.04 (−0.07;−0.01)*
Smoking	Age, education, sex		
Non-smoker		reference	reference
Smoker		0.17 (0.1;0.2)*	−0.03 (−0.06;0.01)
Diagnosis	Age, sex, smoking		
Breast		reference	reference
Gastrointestinal		−0.04 (−0.1;0.1)	−0.01 (−0.04;0.02)
Haematological		0.08 (−0.04;0.2)	−0.03 (−0.06;−0.002)*
Testicular		−0.07 (−0.2;0.3)	−0.1 (−0.2;0.1)
Lung		0.2 (−0.1;0.4)	−0.02 (−0.05;0.02)
Gynaecological		−0.1 (−0.3;0.1)	−0.04 (−0.1;0.1)
Treatment	Age, diagnosis		
No treatment/only surgery		reference	reference
Surgery + chemotherapy		0.02 (−0.03;0.1)	0.01 (−0.02;0.04)
Surgery + radiotherapy		0.03 (−0.03;0.1)	0.01 (−0.02;0.04)
Surgery + radiotherapy + chemotherapy		−0.02 (−0.04;0.08)	0.00 (− 0.03;0.03)
Comorbidity	Age, BMI, smoking, treatment		
No comorbidities		reference	reference
One or more comorbidities		0.01 (−0.02;0.05)	0.00 (−0.02;0.02)
Time since diagnosis	–		
< 12 months		reference	reference
12- < 36 months		−0.1 (−0.2;−0.02)*	−0.02 (−0.04;0.01)
36- < 120 months		−0.1 (−0.2;−0.02)*	0.00 (−0.03;0.02)
120 ≥ months		−0.1 (−0.2;0.01)	0.01 (−0.03;0.05)
Fatigue	Age, BMI, diagnosis, comorbidity, smoking, time since diagnosis, treatment		
< 0.5 SD		−0.01 (−0.05;0.03)	0.02 (−0.003;0.04)
Mean fatigue		reference	reference
> 0.5 SD		0.04 (−0.01;0.08)	0.0 (−0.02;0.03)

*CI* confidence interval, *ref* reference, *SD* standard deviation, * *p* < 0.05, ^#^ the probability of belonging to this activity profile (could be any proportion between 0 and 1) ^a^ based on the DAG in Additional file 1: Figure S1

## Discussion

Based on pooled and harmonized data of 1447 cancer survivors, we found that cancer survivors engage in few minutes of MVPA and spend a large proportion of their day sedentary. We identified six activity profiles with differences in sedentary time, average cpm, time in physical activity bouts, total time in MVPA and time in MVPA bouts. Furthermore, we found that age, gender, weight status, smoking status and fatigue were associated with MVPA and sedentary time whereas age, gender, weight status, smoking status, marital status, cancer type and time since diagnosis were associated with activity profiles.

Results of the current study are in line with results from previous studies reporting sedentary time in survivors of breast (i.e. 66% of accelerometer wear time) [35] and colon (i.e. 61% of accelerometer wear time) [26] cancer. Furthermore, we found that participants spent considerable time (30% of wear time) in sedentary bouts of at least 20 min. Our finding of low MVPA (3% of wear time) confirms previous studies among breast cancer survivors (1%) [35] and Dutch cancer survivors with chronic cancer-related fatigue (6%) [51]. Participants spent on average 26 min per day in MVPA, but only 0.5% of wear time was accumulated in MVPA bouts of 10 or more minutes. Although in some countries, physical activity guidelines highlight the importance of performing MVPA in bouts of at least ten minutes, [39, 52] the 2018 Physical Activity Guidelines now state that any amount of moderate-to-vigorous physical activity may be included in the accumulation of total volume of physical activity and conclude that bouts of any length contribute to health benefits associated with physical activity [53].

The finding of six different activity profiles (i.e. the average profile, the high potential profile, the highly active profile, the highly sedentary profile, the sufficiently active profile and the insufficiently active profile) indicates that classifying cancer survivors’ behaviour based on one dimension of sedentary or active behaviour may be too crude. For example, participants could be categorized based on low sedentary time, but some participants with low sedentary time may also have low levels of MVPA. A recent study investigated activity profiles in patients with chronic cancer-related fatigue and identified three profiles based on accelerometer data of 172 Dutch cancer survivors [51]. Indicators of activity profiles in that study were total sedentary time, physical activity and MVPA, sedentary time in bouts, physical activity and MVPA, and day part distribution (i.e. change score of the average physical activity or sedentary time of two consecutive day parts; morning, afternoon, evening) of sedentary time, physical activity and MVPA. Profiles differed predominantly regarding total physical activity, MVPA, and sedentary time. The identified profiles are generally consistent with profiles identified in our study and indicate that cancer survivors form a heterogeneous group with regard to their physical activity and sedentary time and interventions require a direct goal with respect to each of these behaviours.

Our finding that a younger age and normal weight were associated with higher MVPA, and an older age, obesity and being male were associated with higher sedentary time supports findings of previous studies in cancer survivors [16, 18–20]. Our finding that smoking was associated with lower levels of physical activity supports previous studies among cancer survivors using measures of self-reported physical activity, [54, 55] but contrasted the results of accelerometer assessed physical activity in colon cancer survivors [18]. Smoking status might be correlated with cancer type [56] and the association between smoking status and physical activity and sedentary time might be predominantly present in lung cancer survivors, which were not included in previous studies. Sex was not associated with physical activity time in cancer survivors in previous studies [16, 18–20]. However, multiple studies have investigated correlates of physical activity and sedentary time in sex-specific types of cancer (i.e. breast cancer survivors), making it impossible to investigate whether sex is associated with physical activity or sedentary time [16, 18–20]. Furthermore, we found that lower levels of fatigue were associated with higher levels of physical activity while previous studies did not investigate this association, possibly because fatigue can both be a cause and a result of low physical activity levels [51].

The findings on correlates of activity profiles can help to identify cancer survivors particularly at risk for both an inactive and sedentary lifestyle, and can be used to personalize physical activity interventions by focusing on optimal support for specific (unhealthy) behaviour. For example, it may be advised to increase the intensity of physical activity for survivors with a high potential activity profile or to decrease sedentary time in survivors with an insufficiently active or highly sedentary profile. In contrast to our findings, sex and time since diagnosis have previously not been associated with activity profiles of cancer survivors [51]. Discrepancies between correlates may be explained by differences in: the use of indicators used to define activity profiles, type of accelerometers used (i.e. ProMove 3D, Inertia Technology, The Netherlands versus ActiGraph accelerometers), cut-points used to define sedentary time, physical activity and MVPA, definitions for bouts of sedentary time and number of activity profiles identified by the latent profile analysis.

Strengths of the current study are the large sample size, accelerometer assessed physical activity and sedentary time and uniform measures of these behaviours. We investigated both physical activity and sedentary time in a multinational dataset and we used multiple dimensions of both behaviours to investigate activity profiles of cancer survivors. Furthermore, we used a DAG to identify the minimal adjustment set of possible confounder instead of investigating the role of each variable on the outcome in one model including all possible correlates (and thereby adjusting for all these variables) [50]. Our study has some limitations. First, the DAG used to identify the minimum set of confounders for the association between demographic and clinical characteristics and daily activity was based on current literature and expert opinion, despite the literature revealing inconsistencies with respect to associations between variables, and the direction of the associations. This could have resulted in residual confounding in some of the estimated associations. Second, we investigated activity profiles using latent profile analyses up to six different profiles and BIC was lowest and entropy highest when six profiles were identified. Possibly more activity profiles could be identified based on accelerometer data of participants included in the current study. However, the number of participants fitted in the different profiles would be low and the practical application of small profiles with small differences would be limited. Furthermore, future research should investigate the association between activity profiles and health outcomes to be able to intervene towards optimizing activity profiles for cancer survivors. Third, although using cut-points is the most common method for estimating time spent in different intensities of physical activity, there is some debate in this area. The use of different cut-points (and other data-processing decisions) can result in large variations in estimates of time spent in light-intensity physical activity and MVPA, and alternative methods based on raw acceleration data with machine learning techniques have been proposed [57]. However, the use of cut-points has been and continues to be by far the most common method used to process and analyse accelerometer data [21]. The Freedson cut-points are the most widely applied in this field, and thus allows direct comparison with other studies [21]. These cut-points are based on indirect calorimetry data collected during treadmill activities in a group of university students with a mean age of 24 years [34]. It is likely that the Freedson cut-points underestimate moderate-intensity physical activity in older and less fit individuals. Finally, there is currently no consensus on the definition of a sedentary bout. We defined a sedentary bout as a period of sedentary time of at least 20 min, without allowance for interruptions, whereas different definitions may have been used previously (i.e. ≥ 10 min or > 30 min [58, 59]).

In conclusion, participants in this multinational pooled dataset spent on average only 3% of accelerometer wear time in MVPA and 66% of their time being sedentary. Multiple demographic and clinical characteristics such as age, gender, weight status, smoking status, marital status, fatigue and time since diagnoses were associated with physical activity and sedentary time. These results help to identify cancer survivors particularly at risk for unhealthy activity behaviour. Furthermore, the activity profiles can be used to personalize physical activity interventions for cancer survivors with different activity profiles by focusing on optimal support for specific active or sedentary behaviour.

## Additional file

Additional file 1 **Figure S1.** Directed acyclic graph (DAG) visualizing potential confounders of the association between demographic and clinical characteristics and daily activity. (DOCX 413 kb)

## Data Availability

The datasets used and analysed during the current study are available from the corresponding author on reasonable request.
