# Supplementary material for: Which cancer survivors are at risk for a physically inactive and sedentary lifestyle? Results from pooled accelerometer data of 1447 cancer survivors
Source: Int J Behav Nutr Phys Act. 2019 Aug 16;16:66. doi: 10.1186/s12966-019-0820-7 (PMC6698042; doi:10.1186/s12966-019-0820-7)
Supplement: Supplementary file 1 — Figure S1. Directed acyclic graph (DAG) visualizing potential confounders of the association between demographic and clinical characteristics and daily activity. (DOCX 413 kb) [file 12966_2019_820_MOESM1_ESM.docx]

| 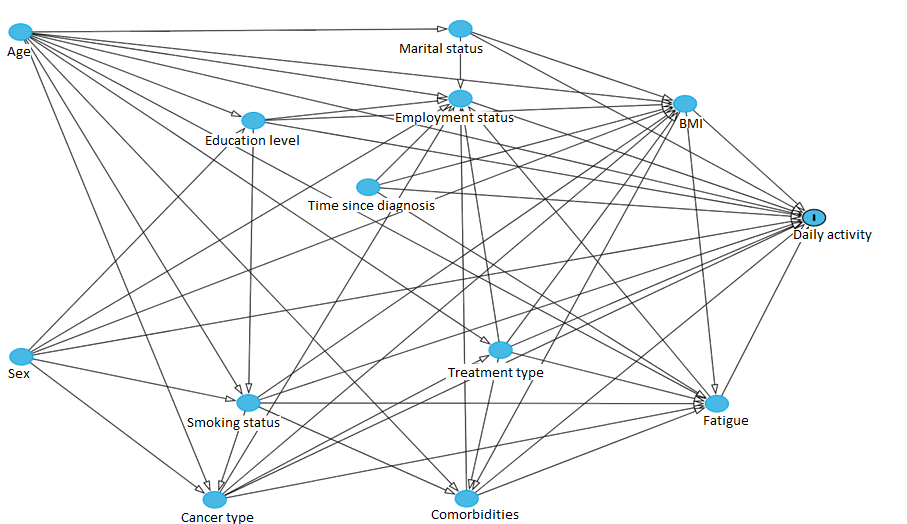 |
| --- |
| **Additional file 1: Figure S1.** Directed acyclic graph (DAG) visualizing potential confounders of the association between demographic and clinical characteristics and daily activity |
